# Weakening AMOC reduces ocean carbon uptake and increases the social cost of carbon

**DOI:** 10.1073/pnas.2419543122

**Published:** 2025-02-24

**Authors:** Felix Schaumann, Eduardo Alastrué de Asenjo

**Affiliations:** ^a^Department of Economics, Center for Earth System Research and Sustainability, University of Hamburg, Hamburg 20146, Germany; ^b^International Max Planck Research School on Earth System Modelling, Max Planck Institute for Meteorology, Hamburg 20146, Germany; ^c^Institute of Oceanography, Center for Earth System Research and Sustainability, University of Hamburg, Hamburg 20146, Germany

**Keywords:** climate change, overturning circulation, carbon cycle, integrated assessment, social cost of carbon

## Abstract

A weakening of the Atlantic Meridional Overturning Circulation (AMOC) has been found to be globally beneficial by economic assessments. This result emerges because AMOC weakening would cool the Northern Hemisphere, thereby reducing expected climate damages and decreasing estimates of the global social cost of carbon dioxide (SCC). There are, however, many other impacts of AMOC weakening that are not yet taken into account. Here, we add a second impact channel by quantifying the effects of AMOC weakening on ocean carbon uptake, using biogeochemically-only coupled freshwater hosing simulations in the Max Planck Institute Earth System Model. Our simulations reveal an approximately linear relationship between AMOC strength and carbon uptake reductions, constituting a carbon cycle feedback that leads to higher atmospheric CO_2_ concentrations and stronger global warming. This AMOC carbon feedback, when incorporated into an integrated climate-economy model, leads to additional economic damages of several trillion US dollars and raises the SCC by about 1%. The SCC increase is similar in magnitude, but of opposite sign, to the SCC effect of Northern Hemisphere cooling. While there are many other potentially relevant economic impact channels, the AMOC carbon feedback alone could thus flip the consequences of AMOC weakening into a net cost to society.

## Significance

The Atlantic Meridional Overturning Circulation (AMOC) is crucial for controlling the state of the Earth system, and is projected to weaken within this century, with potentially dramatic consequences. However, current economic impact studies focus solely on AMOC-related surface cooling, the impact of which is seen as globally beneficial. By quantifying the AMOC-related reduction of ocean carbon uptake, which leads to more atmospheric CO_2_ and more global warming, we find economically negative effects that are not yet accounted for in impact assessments of AMOC weakening. Our study develops projections of the AMOC carbon feedback, estimates the associated economic consequences, and provides a blueprint for how different model types can be combined to comprehensively assess impacts of future AMOC changes.

Assessments of the social cost of carbon dioxide (SCC)—the most important economic impact metric of climate change, which quantifies the economic cost of an additionally emitted ton of CO_2_ (1–4)—have started integrating climate feedbacks and tipping dynamics (5–9). However, a recent expert survey and meta-analysis on the SCC indicates that insufficient representation of Earth system dynamics is still a major driver of underestimated SCC values (10)—an issue also pointed out by the U.S. Environmental Protection Agency (11). We tackle this issue using the example of economic impacts arising from the projected weakening of the Atlantic Meridional Overturning Circulation (AMOC, 12, 13). The AMOC is crucial for regulating the Earth’s climate, and its projected weakening (13–15) could severely impact temperature and weather patterns across the globe (16, 17). Yet, the economic assessment of these impacts has been the source of heated debates (18, 19).

The controversy stems from the fact that existing economic estimates of the impacts of AMOC weakening only take into account direct changes in surface temperature patterns (5, 6). As the AMOC transports large amounts of heat northward, its weakening would lead to large-scale cooling in the Northern Hemisphere alongside warming in the Southern Hemisphere. Pronounced cooling in the Northern Hemisphere locally reduces warming-induced climate damages and thereby decreases estimates of the global SCC (5). But AMOC weakening would have impacts far beyond cooling the Northern Hemisphere (16, 20, 21), for example on precipitation (22), sea levels (23), or the carbon cycle (24–28)—all of which come with large uncertainties and have yet to be incorporated into economic assessments. Here, we focus on adding one key mechanism: the impact of AMOC weakening on ocean carbon uptake. The contribution of this paper is twofold; first, we quantify the impact of AMOC weakening on ocean carbon uptake with Earth system model (ESM) simulations; and second, we assess the effect of carbon uptake reductions on the SCC with an integrated assessment model (IAM).

Carbon uptake is affected by AMOC weakening because the North Atlantic currently takes up a large fraction of anthropogenic CO_2_ (29, 30), facilitated by the AMOC transporting dense and carbon-rich water masses from the surface to the deep ocean (31–33). As the AMOC is projected to weaken due to global warming, this uptake of atmospheric carbon is expected to weaken accordingly (25–28, 34–36). Consequently, more carbon will remain in the atmosphere and increase global warming. We call this the AMOC carbon feedback. The approximate magnitude of the AMOC carbon feedback can be inferred from existing studies that model AMOC weakening alongside an interactive carbon cycle (26–28), but the exact relationship between AMOC strength and the resulting reduction in carbon uptake is still uncertain (37, 38). In order to assess the dynamics and the strength of this relationship, we conduct combined carbon cycle and hosing simulations in the Max Planck Institute Earth System Model (MPI-ESM, version 1.2-LR, 39). Specifically, we combine biogeochemically-only coupled experiments, common in the carbon cycle feedback literature (40–42), with freshwater hosing experiments, common in the AMOC modeling literature (43, 44). Based on these simulations, we establish an approximately linear relationship between AMOC strength and the reduction of ocean carbon uptake.

This reduction in carbon uptake will—through the AMOC carbon feedback—lead to higher atmospheric carbon concentrations and thereby higher global mean temperatures. The rise in global mean temperatures increases expected economic damages from climate change and leads to an increase in the SCC, the economic metric used to quantify the aggregate welfare impact of emitting an additional ton of CO_2_. For estimating how strongly the AMOC carbon feedback affects the SCC, we employ an IAM developed for investigating the economic effects of climate feedbacks and tipping dynamics, called Model for Economic Tipping Analysis (META, 5). We include a modular AMOC carbon component into META, which we calibrate to our quantification of the AMOC carbon feedback, as well as to CMIP6 projections of AMOC strength over the 21st century (13). With this modular setup, we can then assess the economic impact of including the AMOC carbon feedback into META and compare it to the SCC effects of previously studied AMOC-induced surface cooling (5, 6).

**Fig. 1.**
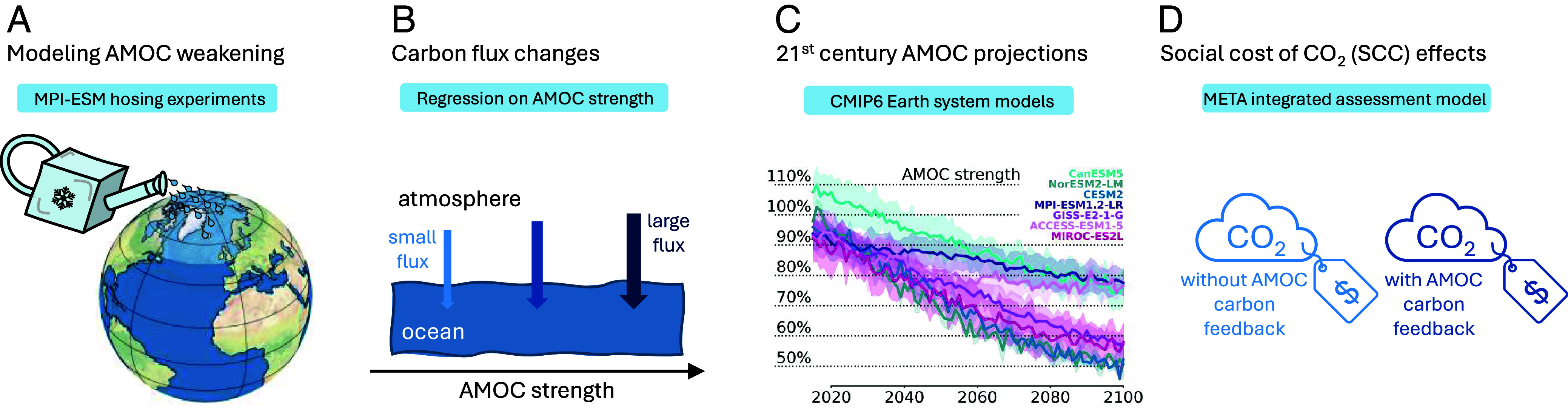
Overview of the modeling approach. (*A*) The MPI-ESM model is used to simulate climate states with an artificially weakened AMOC. (*B*) The resulting changes in annual ocean carbon storage are then analyzed as a function of the simulated AMOC, yielding a quantitative relationship between AMOC strength and changes in carbon fluxes. (*C*) We use AMOC projections from CMIP6 models to estimate the strength of the AMOC carbon feedback until 2100 along the SSP2-4.5 scenario. (*D*) The economic impact of these projected changes is then analyzed in the META IAM, by calculating the relative change in SCC that follows from explicitly incorporating the AMOC carbon feedback into the model structure.

Overall, our approach consists of four elements (Fig. 1). We start by simulating AMOC weakening together with the carbon cycle in MPI-ESM (*A*). We then use these simulations to quantify the dependence of carbon fluxes on AMOC strength (*B*). With this, we proceed to project the strength of the AMOC carbon feedback throughout the 21st century based on CMIP6 models (*C*); and for each of these projections, we finally assess the additional economic damages and the effect on the SCC (*D*).

## Modeling a Weakening AMOC

Studying the carbon cycle effects of AMOC weakening requires ESM simulations with varying AMOC strengths and realistic atmospheric CO_2_ concentrations. Most ESM simulations of AMOC weakening are conducted with preindustrial CO_2_ concentrations, which makes them unsuitable for studying effects on the modern-day carbon cycle (43). While there are ESM simulations of AMOC weakening that include the anthropogenic rise in atmospheric carbon along with the resulting global warming (26, 28), this warming will itself strongly affect ocean circulations. In these simulations, it is therefore difficult to disentangle the carbon cycle effects of AMOC weakening from the carbon cycle effects of global warming. We circumvent this issue by combining freshwater hosing experiments (44) for modeling AMOC weakening with a biogeochemically-only coupled (BGC-only) setup (42) for neutralizing the carbon cycle effects of global warming. In freshwater hosing experiments, the AMOC is artificially weakened by reducing the salinity of the North Atlantic (*Materials and Methods*, 43). This freshening of high-latitude waters is supposed to account for the melting of land ice, which is neglected in ESMs without interacting ice sheets. It reduces the density of water masses in the North Atlantic regions of deep water formation so that less water sinks into the deep ocean and the AMOC as a whole is weakened. BGC-only simulations, on the other hand, are a key element of carbon cycle feedback studies (40, 42). Usually, a fully coupled ESM simulation is compared to a BGC-only simulation, in which the radiative module is decoupled so that rising carbon concentrations have no effect on temperatures. This comparison allows to disentangle temperature effects from other changes affecting the carbon cycle.

We leverage the BGC-only setup for estimating the effects of AMOC weakening on the carbon cycle without picking up temperature-related carbon cycle changes. In contrast to carbon cycle feedback experiments, we do not compare the BGC-only simulations with fully coupled simulations. Instead, we compare a set of BGC-only simulations which vary in AMOC strength. The variation in AMOC strength is generated by applying different magnitudes and spatial patterns of freshwater hosing to BGC-only simulations in MPI-ESM (*Materials and Methods*, 39, 43). Because the rise in carbon concentrations is decoupled from global temperature responses, ocean circulation and the oceanic carbon cycle are only affected by changes in AMOC strength, not by other mechanisms linked to warming. Thereby, we can cleanly identify the effect that a certain amount of AMOC weakening has on carbon uptake in the model.

**Fig. 2.**
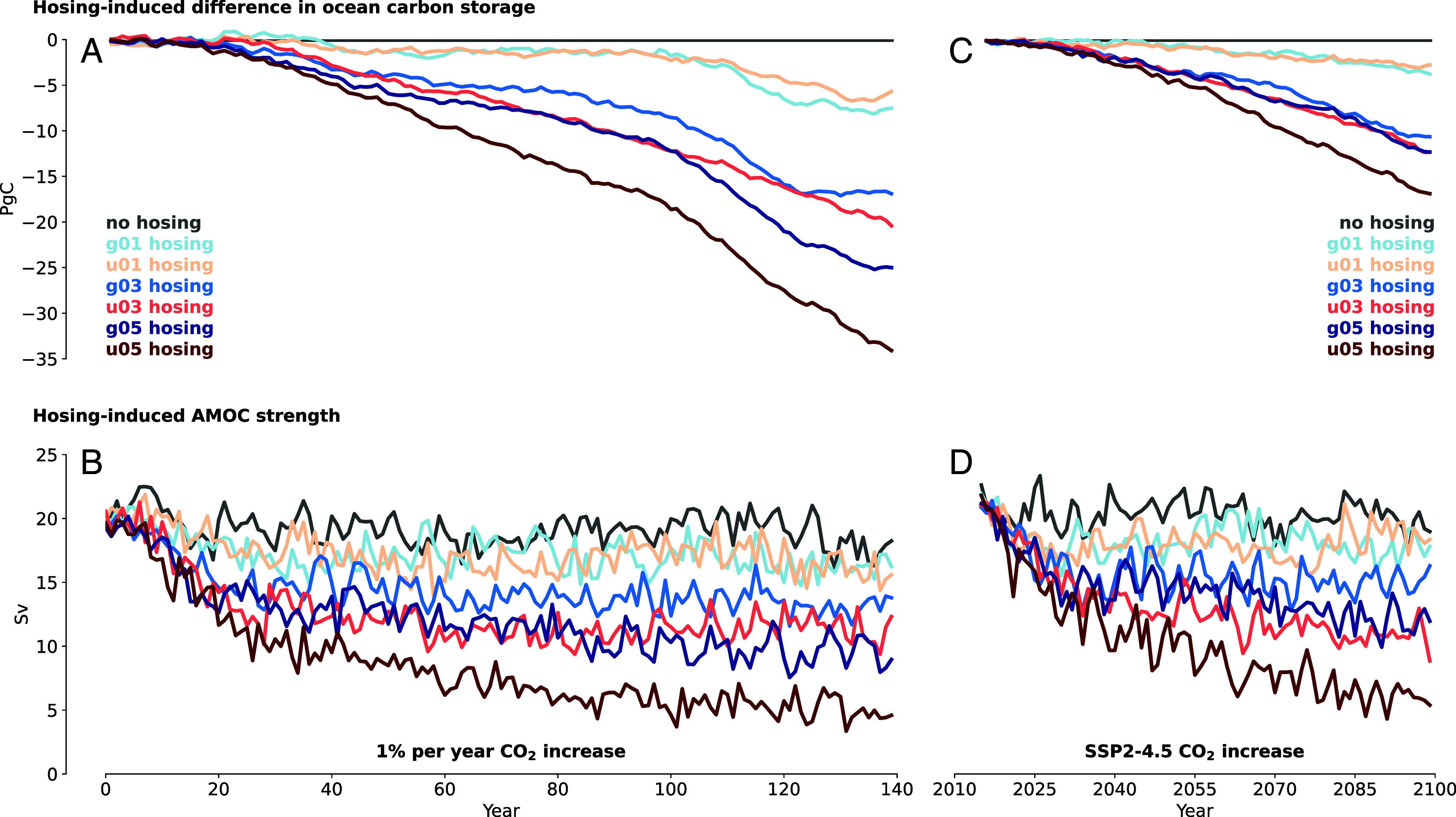
AMOC strength and difference in ocean carbon storage for the 1pct experiment (*A* and *B*) and the SSP2-4.5 scenario (*C* and *D*), as simulated by MPI-ESM. Difference in ocean carbon storage describes the change of ocean carbon storage in a hosing simulation compared to the no-hosing simulation (*Materials and Methods*). The colored labels denote six different hosing configurations: *g* and *u* stand for Greenland and Uniform hosing pattern, whereas *01*, *03*, and *05* denote a freshwater flux of 0.1, 0.3, and 0.5 Sv, respectively (*SI Appendix*, Table S1). Each hosing configuration is applied throughout the modeling period.

We conduct our simulations for two different CO_2_ concentration configurations. As is common in carbon cycle feedback studies, we use an experimental setup where atmospheric CO_2_ concentrations increase by 1% per year, until quadrupling after 140 y. However, this so-called 1pct experiment exhibits much larger CO_2_ concentrations than expected in reality. We therefore repeat our simulations with an SSP2-4.5 scenario of CO_2_ concentrations (45), which can be seen as representing a future of intermediate warming (46) and which is commonly used in SCC research (5). The results from these simulations show that, as expected, the model runs with the weakest AMOC are associated with the lowest ocean carbon storage (Fig. 2). Remarkably, though, in both the 1pct experiment and the SSP2-4.5 scenario, AMOC weakening reduces ocean carbon storage by comparable amounts. This indicates that, at least between 400 and 1,120 ppm, the effect of AMOC weakening on ocean carbon uptake barely depends on the CO_2_ concentration in the atmosphere.

By the end of the modeling period, AMOC weakening leads to changes in ocean carbon storage of more than 30 PgC for the 1pct experiment and more than 15 PgC for the SSP2-4.5 scenario (Fig. 2). To contrast these findings with multimodel evidence, we construct an AMOC-related carbon-climate feedback based on ref. 34, which comprises only those carbon pools and ocean basins for which the feedback strength correlates significantly with the AMOC strength across models (*Materials and Methods*). For strong AMOC weakening of 15 Sv, comparable to the u05 hosing run, this results in carbon storage reductions of 32 PgC at the end of the 1pct experiment. Thus, carbon-climate feedback estimates that harness variation of AMOC strength among CMIP6 models yield very similar results to our 1pct hosing experiments in MPI-ESM. The effects of AMOC weakening on ocean carbon storage in SSP scenarios are also estimated by Boot et al. (28), who find carbon storage reductions of between 7.5 and 15 PgC by 2100. Importantly, though, they compare hosing simulations against fully coupled simulations, which already exhibit a weakened AMOC. The resulting carbon storage reductions are thus only a partial estimate of the overall effect of AMOC weakening. Nevertheless, these other two estimates—both of which come from ESM simulations where AMOC weakening occurs alongside global warming—corroborate the effect size of AMOC-related carbon storage reduction that results from our own simulations in MPI-ESM.

## AMOC-Induced Carbon Flux Changes

**Fig. 3.**
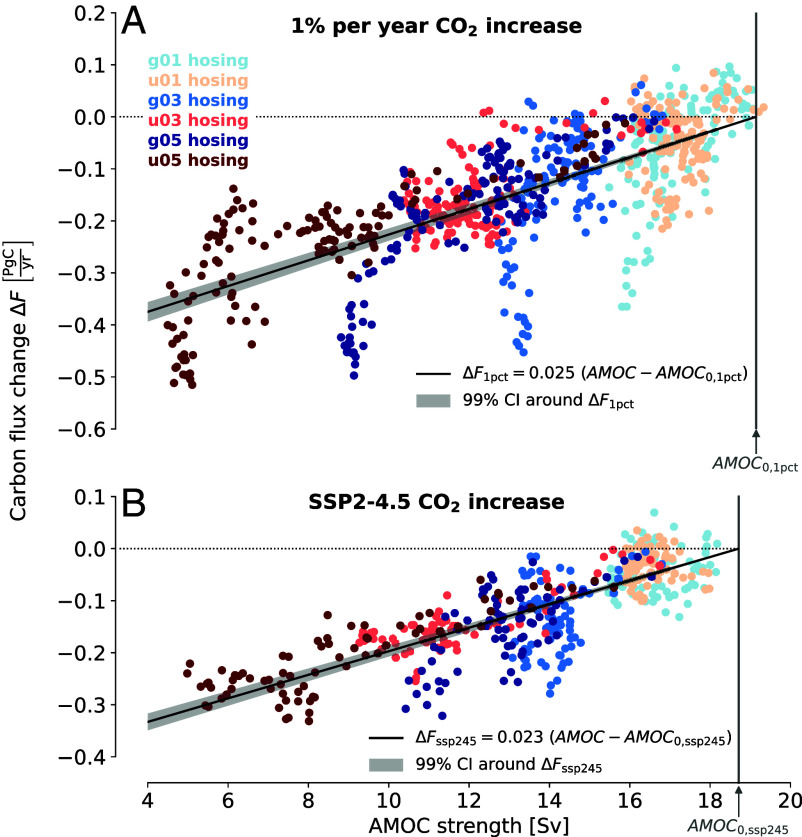
Carbon flux changes as a function of AMOC strength for the 1pct experiment (*A*) and for the SSP2-4.5 scenario (*B*). Both the AMOC strength and the carbon flux time series are smoothed with a 10-y running mean (*Materials and Methods*, *SI Appendix*, Fig. S1 for different running mean windows). Vertical gray lines denote the mean AMOC strength of the simulation without hosing. Black lines are linear regressions with zero intercepts (*Materials and Methods*), and gray shading is the 99% CI of the regression coefficient.

Beyond the overall size of the effect on end-of-century ocean carbon storage, the dynamics of AMOC weakening and carbon storage matter—that is, how exactly the change in carbon storage in a given year depends on the AMOC strength. In order to quantify this relationship, we compute year-on-year changes in ocean carbon storage, which we refer to as carbon fluxes, for each simulation. By subtracting carbon fluxes in the simulation without hosing from carbon fluxes in the hosing simulations, we can define the change in carbon fluxes that is directly caused by hosing—which we call the AMOC-induced carbon flux change. We find that there is an approximately linear relationship between the AMOC strength in a given year and the resulting change of carbon fluxes into the ocean (Fig. 3). For illustration, if the AMOC strength is 10 Sv, the yearly carbon flux into the ocean is reduced by about 0.2 PgC, compared to an AMOC of preindustrial strength (19 Sv).

To capture the emerging relationship between AMOC strength and carbon flux changes, we perform a linear regression for the 1pct experiment and the SSP2-4.5 scenario (Fig. 3 and *SI Appendix*, Table S2). The regression coefficient is slightly higher for the 1pct experiment than for the SSP2-4.5 scenario ($0.025\frac{\;\text{Pg}}{\;\text{yr Sv}}$ vs. $0.023\frac{\;\text{Pg}}{\;\text{yr Sv}}$), but the results are remarkably similar. This provides further evidence that, under climate change, carbon fluxes into the ocean do not seem to be limited by atmospheric CO_2_ concentrations, but rather by the transport of carbon-rich upper-ocean water masses into the deep ocean. Because the SSP2-4.5 scenario of CO_2_ concentrations is more realistic than the 1pct experiment, as well as for the sake of being conservative and consistent with the SCC literature, we use the SSP2-4.5 coefficient of $0.023\frac{\;\text{Pg}}{\;\text{yr Sv}}$ for further calculations. With this relationship at hand, we can now proceed to 21st-century projections of AMOC weakening, whereby every annual value of AMOC strength can be associated with a change of carbon fluxes into the ocean.

## Projecting the AMOC Carbon Feedback

**Fig. 4.**
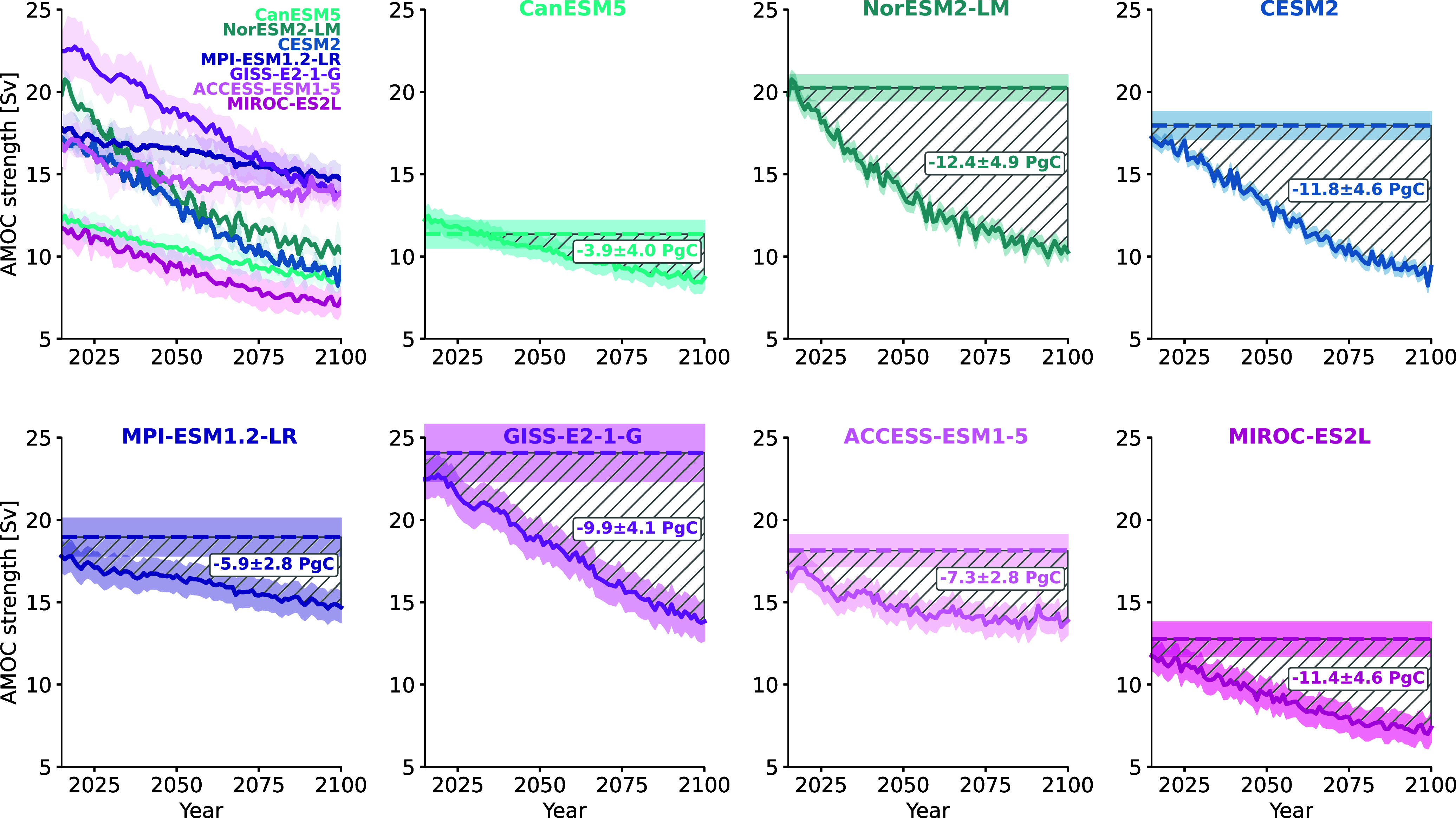
Projected reductions in ocean carbon storage. The *Top-Left* panel shows all seven SSP2-4.5 ESM projections for AMOC strength that are used in this paper. Each of them has at least three ensemble members; the thick lines represent the ensemble mean and the shading the ensemble SD. The other panels show one ESM projection each, together with the preindustrial strength of the respective ESM. For the preindustrial AMOC strength, the dotted line represents the mean strength and the shading one SD. The difference between projected AMOC strength and preindustrial AMOC strength governs the strength of carbon flux change, so the hatched area symbolizes the cumulative amount of carbon that is not stored in the ocean by 2100. The labels on the hatched areas show this carbon storage reduction in 2100, calculated with 10,000 Monte Carlo runs, with the uncertainty range corresponding to one SD. Note that the reductions in ocean carbon storage are not exactly proportional to the size of hatched areas in this plot, because we model carbon flux changes as depending on relative AMOC weakening compared to preindustrial AMOC strength, not on absolute AMOC weakening as portrayed here.

CMIP6 models project a substantial amount of AMOC weakening along the SSP2-4.5 scenario, but both initial AMOC strengths and amounts of weakening vary strongly across models (Table 1 and Fig. 4). We compare CMIP6 projections based on their relative AMOC weakening compared to preindustrial AMOC strength and use them to calibrate a purpose-built AMOC carbon component of the META model. In contrast to the existing AMOC component in META, we do not define a probability for stochastic AMOC weakening, but instead include an explicit representation of AMOC strength, which can be calibrated to match projections by CMIP6 models (*Materials and Methods*). Hereby, we make sure to only use CMIP6 models with several ensemble members so that we can account for internal variability in our projections (47). In our own projections with the META model, we use the internal variability of CMIP6 ensemble projections as well as a constrained parameter set of the FaIR climate emulator (48) to obtain 10,000 Monte Carlo parameter samples (*Materials and Methods*).

**Table:** Table 1.

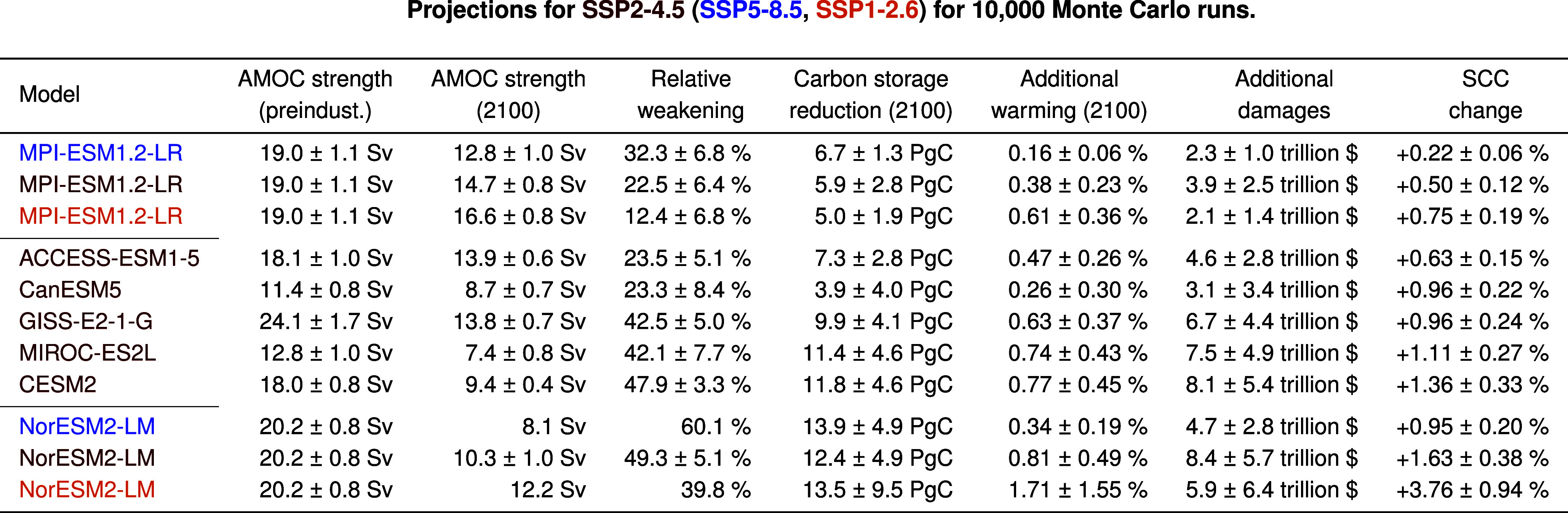

By the end of the century, the AMOC weakens between 20 and 50% in our projections along the SSP2-4.5 scenario (Table 1). As a result of this AMOC weakening, the ocean carbon storage in 2100 is reduced by between 3.9 and 12.4 PgC (Fig. 4). For comparison, yearly CO_2_ emissions from fossil fuels are currently at around 10 PgC (49). The projected reduction in ocean carbon storage corresponds to additional carbon in the atmosphere (50), which affects global temperatures. This additional warming due to the AMOC carbon feedback is small, as it leads to relative global temperature increases between 0.26% and 0.81% by 2100 (Table 1). Nevertheless, even this slight increase in global temperatures has a non-negligible impact on economic damages from climate change. We aggregate the discounted future climate damages that occur between 2015 and 2200 due to the AMOC carbon feedback with the damage function calibrated to (51, *Materials and Methods*). This yields projected additional damages of between 3.1 and 8.4 trillion US dollars (Table 1). Beyond a projected increase in expected climate damages, however, the AMOC carbon feedback also affects the main metric with which the societal damage from an additional ton of CO_2_ emissions is estimated: the SCC.

## Effects on the SCC

**Fig. 5.**
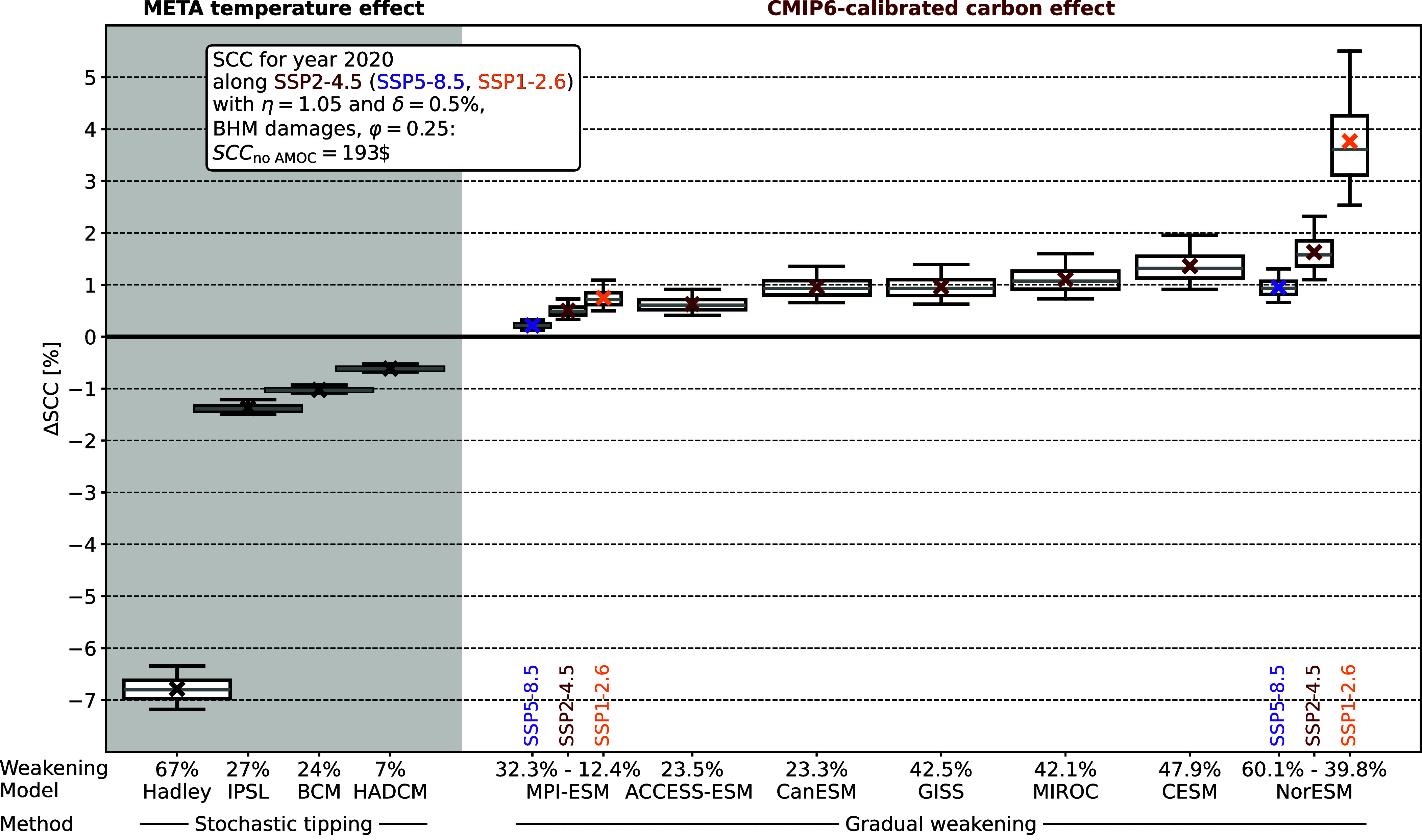
Percentage change in SCC due to AMOC effects. In the gray panel (*Left*), the relative SCC effects of AMOC-induced surface cooling are reproduced based on ref. 5. In the white panel (*Right*), the relative SCC effects of the AMOC carbon feedback are estimated based on different 21st-century AMOC projections by CMIP6 models. Stochastic tipping refers to the way in which the AMOC component is implemented in the META model, whereby the probability of tipping increases with global temperatures and, once the AMOC is tipped, surface temperature patterns change over the course of 35 y (5). On the other hand, gradual weakening refers to our approach of calibrating AMOC scenarios to CMIP6 models, whereby the modeled AMOC strength exactly follows ESM projections along a given emission scenario. AMOC weakening describes the relative AMOC decrease in the tipped state (stochastic tipping) or the relative AMOC decrease between preindustrial conditions and 2100 (gradual weakening). Boxes show interquartile ranges, with medians in gray. Crosses mark the mean effect, and whiskers show the 5th and 95th percentile. Each box covers a distribution of results that emerge from 10,000 Monte Carlo results, with uncertainty in the FaIR climate module, as well as about preindustrial AMOC strength, AMOC projections, and the coefficient linking AMOC strength to reduced carbon uptake. We show the full distribution of 10,000 Monte Carlo samples—versions with 0.1% or 1% of results trimmed at each tail are shown in the (*SI Appendix*, Fig. S5). The text box on the *Top Left* lists important parameters for the SCC calculation, with η denoting the elasticity of intertemporal substitution, δ the rate of pure time preference, and φ the persistence parameter (*Materials and Methods*, 5). BHM damages refer to the damage function based on ref. 51.

The SCC increase of including the AMOC carbon feedback based on SSP2-4.5 projections lies between 0.5% and 1.63% (Table 1), when using the welfare and persistence parameters shown in the box of Fig. 5. SCC effects are estimated by calculating the SCC in META for both the base setup and a setup with AMOC carbon feedback and then calculating the relative change in percent (*Materials and Methods*). The results are robust to assumptions about the spatial pattern of warming; because AMOC weakening will impact surface temperature patterns, we have also estimated these SCC effects against the backdrop of different AMOC-induced temperature patterns. We used the same four spatial patterns as previous studies on the subject (5, 6), which go back to transient hosing simulations under greenhouse gas forcing (52, 53), but none of these AMOC-induced temperature patterns affect relative SCC changes caused by the AMOC carbon feedback (*SI Appendix*, Table S3). Contrasting the impacts of the AMOC carbon feedback with the previously studied AMOC cooling effects (Fig. 5, *Left*; 5) shows that the SCC effects of the AMOC carbon feedback are similar in magnitude, but of opposite sign, to the SCC effects of AMOC-induced cooling.

For the AMOC carbon feedback, there is a stable relationship between the amount of AMOC weakening that a certain model projects and the resulting rise in SCC estimates (Table 1). This relationship, however, breaks down when comparing changes along different socioeconomic scenarios. For the two models with weakest and strongest SCC effect, respectively, we also calculated the SCC effect of the AMOC carbon feedback along SSP5-8.5 and SSP1-2.6 (Fig. 5). For both models, the SCC increase is diminished under a high-emission scenario and magnified under a low-emission scenario. This disproportionate SCC effect of AMOC changes under low-emission scenarios is caused by the fact that projections of AMOC strength along different SSP scenarios are barely distinguishable until late in the 21st century (13). At the same time, global warming levels differ strongly across these scenarios. Therefore, relative to the amount of global warming, AMOC weakening is much more pronounced in low-emission scenarios (Table 1). Hence, the AMOC carbon feedback becomes more economically relevant the more the world moves toward limiting global warming to internationally agreed levels.

## Discussion

We provide estimates of the AMOC carbon feedback and find an approximately linear relationship between AMOC strength and carbon flux changes. This allows us to make projections for a range of CMIP6 models, whose ScenarioMIP output currently does not capture the AMOC carbon feedback because of being forced by CO_2_ concentrations instead of CO_2_ emissions (54). As a second contribution, we incorporate this relationship into an IAM, with which we explore the economic impacts of the AMOC carbon feedback. This yields SCC changes of a magnitude that could flip economic impact assessments of AMOC weakening from a global benefit into a global cost. Both parts of our study are subject to uncertainties and limitations. The estimates of AMOC-induced carbon flux changes rest on our simulations with the MPI-ESM model. Some features of the ocean and carbon cycle dynamics would likely be different in equivalent simulations with other ESMs, for example the late intensification of carbon uptake reductions in the 1pct experiment (Fig. 3 and *SI Appendix*, Fig. S2). This implies that the CI of our regression coefficient might not be capturing the full amount of uncertainty. Nevertheless, the fact that multimodel evidence from CMIP6 carbon cycle feedback experiments as well as other hosing-based studies (28) yield similar results increases confidence in the dynamics and magnitude of our findings. The economic impact estimation is also subject to uncertainties as well as normative and structural modeling choices (10). As demonstrated by many studies, the SCC is very sensitive to choices of discounting and damage function parameters (4, 55–58). Our absolute SCC values, as well as the additional damages we estimate, are equally sensitive to these parameter choices (*SI Appendix*, Table S4). For this reason, we focus on the relative change in SCC as our main outcome variable. While the absolute SCC value can change drastically, the relative SCC impact of including the AMOC carbon feedback is more robust to alternative discounting and damage function parameters (*SI Appendix*, Table S4).

Of all the possible impacts of AMOC weakening, the literature has so far only included a single one: the change in surface temperature patterns, which is dominated by Northern Hemisphere cooling. Here, we add a second important mechanism that acts through the impact of AMOC weakening on the carbon cycle. By considering a single additional mechanism through which AMOC weakening might affect the economy, we find SCC changes that might flip the sign of the overall effect. But there are many more potential impacts of AMOC weakening, such as changes in precipitation, sea level, or North Atlantic storm tracks. Therefore, our results should not be interpreted as an impact estimate of AMOC weakening as such, but rather as the second step of a long journey toward assessing the full economic consequences of AMOC weakening. Recent studies have been warning about the possibility of a complete AMOC collapse (14, 59, 60), a scenario which we do not cover in this study. Our results are conservative, as they are based on SSP2-4.5 AMOC projections from CMIP6 models—none of which feature AMOC collapse. Using the same scenario across all parts of this study is important for the internal consistency of our results, but it should be noted that, in the event of stronger AMOC weakening than projected by current models, SCC impacts would increase considerably.

## Materials and Methods

### ESM Simulations.

#### Freshwater hosing.

We perform freshwater hosing experiments in the MPI-ESM1.2-LR model (39). There are two common ways of introducing the freshwater flux (implemented as a negative salinity flux) in these experiments (43): either as a uniform field across the whole Atlantic north of 50Â°N or as a field around Greenland that decays exponentially with distance to the coast. While Greenland hosing is arguably the more realistic setup, uniform hosing fields are more common in the literature; we therefore run both types of hosing patterns. For both patterns, we use different hosing strengths, corresponding to freshwater fluxes of 0.1, 0.3, and 0.5 Sv, respectively. Given that 0.1 Sv is already on the higher end of estimates of expected input from future Greenland ice sheet melting, these hosing experiments should not be interpreted as a realistic freshwater forcing, but rather as a means for generating internally consistent model worlds that span a wide range of AMOC strengths.

#### CO_2_ forcing.

In contrast to the hosing simulations studied in ref. 43, which are conducted against the background of preindustrial climate conditions, we apply anthropogenic CO_2_ forcing. We do that in two different experimental setups. In the first setup, we run one set of simulations where atmospheric CO_2_ concentrations increase by 1% per year, in accordance with the literature on carbon cycle feedbacks (41). In the second setup, we prescribe more realistic atmospheric CO_2_ concentrations, following a historical simulation first and branching into the SSP2-4.5 emission scenario from 2015 until 2100 (45). The hosing for the SSP2-4.5 setup is only applied after 2015. All the conducted ESM simulations are listed in *SI Appendix*, Table S1.

#### Biogeochemical-only coupling.

Our simulations feature freshwater forcing and CO_2_ forcing, both of which affect the oceanic carbon cycle; the freshwater forcing by reducing water mass density and weakening the AMOC, and the CO_2_ forcing by causing global warming which again causes changes in, among others, solubility, carbonate chemistry, density, mixed layer depth, and biological activity. For this study, we are exclusively interested in carbon cycle changes caused by AMOC-related reductions in physical water mass transport into the deep ocean. All warming-induced carbon cycle changes are potential confounders for this analysis, which we address by running biogeochemically-only coupled (BGC-only) simulations. BGC-only simulations artificially decouple atmospheric CO_2_ concentrations from the model’s radiation component so that there is no warming impact from CO_2_. This ensures that the ocean carbon cycle is not affected by CO_2_-induced warming, but only by the AMOC changes we induce through freshwater hosing. At the same time, the carbon cycle as such is undisturbed, and with the 1pct experiment and the SSP2-4.5 scenario, we can study how different amounts of atmospheric CO_2_ concentrations influence carbon fluxes into the ocean and how these depend on AMOC strength.

#### AMOC-related carbon-climate feedback.

A second method for estimating the influence of AMOC strength on ocean carbon uptake takes the overall oceanic carbon-climate feedback (40) and disentangles it by ocean basin and by carbon pool in order to determine which parts of the carbon-climate feedback depend on AMOC weakening (34).

Three carbon-climate feedback is differentiated by three carbon pools: saturated carbon, which reflects changes in solubility governed by warming and carbonate chemistry; disequilibrium carbon, which reflects changing amounts of water masses sinking before reaching chemical equilibrium with the atmosphere; and regenerated carbon, which reflects changes in biological processes and residence times of water masses in the ocean interior. Katavouta and Williams (34) find that the carbon-climate feedbacks of the Atlantic disequilibrium carbon pool ($\gamma_{\text{Atl, dis}}$) and the carbon-climate feedbacks of the Atlantic regenerated carbon pool ($\gamma_{\text{Atl, reg}}$) across different CMIP6 models both correlate with the amount of AMOC weakening that the respective model exhibits.

We harness this correlation to construct an AMOC-related carbon-climate feedback $\gamma_{\text{AMOC}}$

[1] $$\begin{array}{c} \gamma_{\text{AMOC}}=\gamma_{\text{Atl, dis}}+\gamma_{\text{Atl, reg}}. \end{array}$$

Regressing this on AMOC strength at the end of the carbon cycle feedback experiments yields the following equation (*SI Appendix*, Fig. S3):

$$\begin{array}{c} \gamma_{\text{AMOC}}=-0.58\frac{\;\text{PgC}}{\;\text{SvK}}\left(\Delta AMOC-3.84\;\;\text{Sv}\right). \end{array}$$

To calculate end-of-simulation ocean carbon storage reductions, we need to choose a value for AMOC weakening ΔAMOC and multiply $\gamma_{\text{AMOC}}$ with global mean surface temperature at the end of the simulation, as carbon-climate feedback values are normalized with respect to temperature change. From ref. 40, we get $T_{\text{1pct}}\left(140\right)=4.87\;\text{K}$, which simplifies the AMOC-related ocean carbon storage reduction $\Delta C_{\text{AMOC}}$ to

[2] $$\begin{array}{cc} \Delta C_{\text{AMOC}}\left(\Delta AMOC\right) & =-\gamma_{\text{AMOC}}\left(\Delta AMOC\right)\cdot T_{\text{1pct}}\left(140\right) \end{array}$$

[3] $$\begin{array}{cc} & =2.825\frac{\;\text{PgC}}{\;\text{Sv}}\left(\Delta AMOC-3.84\;\;\text{Sv}\right). \end{array}$$

#### Analyzing AMOC strength and ocean carbon storage.

We define AMOC strength as the maximum meridional flow of water at 26.5^°^N in the Atlantic Ocean for all depths z and integrated from west to east:

[4] $$AMOC(t)=\underset{z}{\max}\left\{\int_{W_{Atl}}^{E_{Atl}}\int_{0}^{z}v\left(lat=26.5^{{}^{\circ}}\;\text{N},lon,z\right)\;\;\text{d}z\;\;\text{d}lon\right\},$$

where v(lat,lon,z) is the meridional northward velocity of water at a specific point in the ocean.

We define global ocean carbon storage as the volumetric integral of dissolved inorganic carbon (DIC) concentrations:

[5] $$\begin{array}{c} C_{\text{ocean}}\left(t\right)=c\int_{V}DIC\left(t\right)\;\;\text{d}V, \end{array}$$

with $c=12.01\frac{\;\text{g}}{\;\text{mol}}$.

For both of these quantities, we extract annual values for all MPI-ESM simulations, which form the basis of further calculations. The annual dataset of AMOC strength and carbon storage is available as part of our open-access code repository.

#### AMOC-induced carbon flux changes.

We define yearly global carbon fluxes F(t) as the year-on-year change in $C_{\text{ocean}}\left(t\right)$. In order to investigate the impact of different AMOC strengths on carbon fluxes into the ocean, we consider the difference between yearly carbon fluxes in hosing simulations and yearly carbon fluxes in the respective simulation without hosing; we call this quantity the AMOC-induced carbon flux change $\Delta F_{\text{AMOC}}\left(t\right)$

[6] $$\begin{array}{c} \Delta F_{\text{AMOC}}\left(t\right)=F_{\text{hosing}}\left(t\right)-F_{\text{no hosing}}\left(t\right). \end{array}$$

To relate the carbon flux change $\Delta F_{\text{AMOC}}\left(t\right)$ to the amount of AMOC weakening, we plot it as a function of AMOC(t), as in Fig. 3. For both $\Delta F_{\text{AMOC}}$ and AMOC, we apply a 10-y running mean in order to capture the overall dynamics rather than short-term fluctuations in carbon storage and AMOC strength. The same results for different running mean windows are shown in the *SI Appendix*, Fig. S1. We then perform two linear regressions, which we call $\Delta F_{0}$ and $\Delta F_{1}$:

[7a] $$\begin{array}{c} \Delta F_{0}=0+c_{0}\cdot \left(AMOC-AMOC_{0}\right) \end{array}$$

[7b] $$\begin{array}{c} \Delta F_{1}=c_{1}+c_{2}\cdot \left(AMOC-AMOC_{0}\right), \end{array}$$

where $AMOC_{0}$ is the average AMOC strength of the simulations without hosing.

The difference between the two regressions is that $\Delta F_{0}$ has no 0th-order term so that the AMOC-induced carbon flux change vanishes by definition if the AMOC strength is unchanged. For internal consistency, this is a desirable property. Given that the linear regression coefficients $c_{0}=0.023\frac{\;\text{PgC}}{\;\text{yr}\;\;\text{Sv}}\left(=0.025\frac{\;\text{PgC}}{\;\text{yr}\;\;\text{Sv}}\right)$ and $c_{2}=0.022\frac{\;\text{PgC}}{\;\text{yr}\;\;\text{Sv}}\left(=0.026\frac{\;\text{PgC}}{\;\text{yr}\;\;\text{Sv}}\right)$ for the SSP2-4.5 scenario (the 1pct experiment) are very similar (*SI Appendix*, Table S2), we use $\Delta F_{0}$ for further calculations.

### Impacts of the AMOC Carbon Feedback.

#### Integration into META IAM.

We use the most recent version of the META IAM (5) for assessing the 21st-century impacts of the AMOC carbon feedback on carbon storage, temperature, climate damages, and the SCC. The META model has a base configuration that comprises socioeconomic and emission scenarios, the FaIR 2.0.0 simple climate model (48), pattern-scaling, a country-level damage function, and a discounted utility component. On top of that, META has several modules for tipping dynamics and climate feedbacks, among them the AMOC-induced cooling effect. We build an AMOC carbon module that differs from the existing AMOC cooling module in that it does not follow stochastically triggered tipping dynamics, but instead comes with an explicit representation of yearly AMOC strength, which again depends on global temperatures. We introduce the variable ΔAMOC, which describes AMOC weakening and depends on the change in atmospheric temperatures since the beginning of the scenario modeling period in 2015:

[8] $$\begin{array}{c} \Delta AMOC\left(t\right)=\beta_{\text{AMOC}}\left(t\right)\left(T_{\text{atm}}\left(t\right)-T_{\text{atm}}\left(2015\right)\right). \end{array}$$

The parameter $\beta_{\text{AMOC}}$ describes the sensitivity of the AMOC to global warming. For stylized scenario analyses, this parameter can be taken to be constant, but it can also be calibrated such as to follow annual projections of AMOC strength. Note that ΔAMOC is positive when the AMOC weakens.

Absolute AMOC strength AMOC is obtained by

[9] $$\begin{array}{c} AMOC\left(t\right)=AMOC\left(2015\right)-\Delta AMOC\left(t\right)+\epsilon_{\mathrm{AMOC}}\left(t\right), \end{array}$$

where $\epsilon_{\mathrm{AMOC}}$ is a random fluctuation in AMOC strength that can be calibrated to AMOC projection uncertainty.

The AMOC-induced reduction in carbon uptake is formally equivalent to additional emissions, which we model through the AMOC-induced carbon flux change $\Delta F_{\text{AMOC}}$

[10] $$\begin{array}{c} \begin{array}{cc} \Delta F_{\text{AMOC}}\left(t\right)= & \left(c_{0}+\epsilon_{c_{0}}\right)\cdot (AMOC_{\text{pi}}+\epsilon_{AMOC_{\text{pi}}}\\ & -AMOC\left(t\right))\cdot \frac{AMOC_{\text{pi,MPI}}}{AMOC_{\text{pi}}}. \end{array} \end{array}$$

Here, $c_{0}$ relates AMOC strength to carbon flux changes (Eq. **7a**, *SI Appendix*, Table S2), and $\epsilon_{c_{0}}$ represents the uncertainty about $c_{0}$. $AMOC_{\text{pi}}$ is the preindustrial AMOC strength of a given model that the AMOC carbon module is calibrated to, and $\epsilon_{AMOC_{\text{pi}}}$ the associated uncertainty. The second term in Eq. **10** describes AMOC weakening in year t, and the third term converts the weakening into equivalent weakening in MPI-ESM. We model the carbon flux change as dependent on the AMOC weakening with respect to preindustrial values, not 2015 values, because in our MPI-ESM simulations, we compare hosing-induced changes to no-hosing simulations that do not exhibit any warming; the appropriate reference is thus the preindustrial AMOC strength. Since our estimates of $c_{0}$ are obtained with MPI-ESM and absolute AMOC weakening differs widely between ESMs, we convert model-specific weakening into equivalent weakening in MPI-ESM through the ratio of respective preindustrial AMOC strengths. The additional CO_2_ flux $\Delta F_{\text{AMOC}}$ is then passed on to the emissions module of META, from where it influences global temperatures and climate damages.

#### Calibration to CMIP6 AMOC projections.

The AMOC carbon module of META can be calibrated to stylized scenarios of AMOC weakening, but also to ESM projections. We collected those historical and SSP2-4.5 simulations by ESMs participating in CMIP6 that are available from the Earth System Grid Federation (ESGF) node of the German Climate Computing Center (DKRZ) and include the variable msftmz for calculating AMOC strength. Of these, we use only the ones that have at least three ensemble members. For MPI-ESM1.2-LR and NorESM2-LM, we additionally collected SSP5-8.5 and SSP1-2.6 AMOC projections; here, we have only one ensemble member of NorESM2-LM and hence no ensemble SD. We also collected preindustrial control simulations of all the models for which we have AMOC projections. From these preindustrial control simulations, we get the mean and SD of preindustrial AMOC strength.

For calibrating the AMOC carbon component, we first extract the time series for global mean surface temperature from META along the SSP2-4.5 scenario. Then we choose $\beta_{\text{AMOC}}\left(t\right)$ such that for every year, the deterministic SSP2-4.5 temperature scenario leads to AMOC weakening that exactly corresponds to a given ESM projection of AMOC strength. This implies that for warming scenarios above (below) the deterministic SSP2-4.5 in META, the AMOC will weaken more (less) than projected by a given ESM.

#### Uncertainty treatment.

For all projections and SCC calculations, we run 10,000 Monte Carlo samples. For this, we sample parameters for the simple climate model FaIR from the constrained parameter ensemble in ref. 48, following the implementation in ref. 61.

To calibrate the uncertainty about the preindustrial AMOC strength of a given ESM, we use the SD of the distribution of AMOC strength in the model’s historical experiment. For every Monte Carlo sample, we draw from a normal distribution with this SD and use this draw as the value for $\epsilon_{AMOC_{\text{pi}}}$. To calibrate the uncertainty about AMOC projections, we average the ensemble SD over time and then, for every year, draw from a normal distribution with this SD. This gives a time series of $\epsilon_{\mathrm{AMOC}}\left(t\right)$, which is able to emulate the year-on-year fluctuations that we observe in both projections and observations of AMOC strength. To calibrate the uncertainty about the carbon flux change resulting from a certain AMOC strength value, we take the SE of the regression coefficient $c_{0}$ in Eq. **7a** and for every Monte Carlo sample, we draw an $\epsilon_{c_{0}}$ value from a normal distribution.

#### Economic damage estimation.

To calculate additional climate damages caused by the AMOC carbon feedback, we extract the path of total climate damages from each Monte Carlo simulation of the META model and then take the difference between total damages in a model configuration with the AMOC carbon feedback and a model configuration without the AMOC carbon feedback. This time series of additional damages is aggregated and discounted to 2015 net present values, which is when the SSP2-4.5 scenario, and hence the AMOC weakening, starts. We use a social discount rate based on the deterministic Ramsey rule, with elasticity of marginal utility of consumption η=1.05 and rate of pure time preference δ=0.5% as in META, and the global growth rate as projected by the SSP2-4.5 scenario. Because the META model variables for consumption and damages are given in 2010 US dollar values, we adjust them to 2024 US dollar values by multiplying with 1.44 based on inflation rates.

For all economic calculations, we retain the parameter values of the most recent version of META (Github, 5); the values are also given in the text box of Fig. 5. The damage persistence parameter φ describes the amount of climate damage that is still felt in the year after the damage is caused. A φ value of 1 means there is no persistence; a φ value of 1 means there is full persistence. The value φ=0.25, as used in the META model, is calibrated based on ref. 62. The results of a sensitivity analysis on η, δ, φ, and the damage function coefficient are shown in the *SI Appendix*, Table S4.

#### Assessing SCC changes.

The SCC effect of the AMOC carbon feedback is estimated in the base META model, without any further feedbacks or tipping dynamics activated. Since META does not optimize, this SCC estimate is contingent on the underlying socioeconomic scenario, in our case SSP2-4.5 (and for two AMOC projections also SSP5-8.5 and SSP1-2.6). We always report values for the SCC in 2020.

We determine the relative SCC impact of including the AMOC carbon feedback through

[11] $$\begin{array}{c} \Delta SCC=\frac{SCC_{\text{AMOC}}-SCC_{\text{no AMOC}}}{SCC_{\text{no AMOC}}}. \end{array}$$

We always compare the base META model (“no AMOC") and the META model with the activated AMOC carbon feedback (“AMOC") for the same Monte Carlo sample so that the difference between these two values stems only from the inclusion of the AMOC carbon feedback, not from uncertain parameters that differ between runs. This results in 10,000 estimates of the relative SCC effect ΔSCC of the AMOC carbon feedback, which are depicted in Fig. 5.

## Supplementary Material

Appendix 01 (PDF)

## Data Availability

Earth system model simulations, integrated assessment model simulations, model code, and analysis scripts have been deposited at https://github.com/felixschaumann/AMOC-Carbon (https://doi.org/10.5281/zenodo.14290084) (63). The modified version of the META model that is used to integrate the AMOC carbon feedback can be found here https://doi.org/10.5281/zenodo.14290001 (64). All other data are included in the manuscript and/or *SI Appendix*.
