# Supplementary material for: Weakening AMOC reduces ocean carbon uptake and increases the social cost of carbon
Source: Proc Natl Acad Sci U S A. 2025 Feb 24;122(9):e2419543122. doi: 10.1073/pnas.2419543122 (PMC11892582; doi:10.1073/pnas.2419543122)
Supplement: Supplementary file 1 — Appendix 01 (PDF) [file pnas.2419543122.sapp.pdf]

PNAS

# Supporting Information for

## Weakening AMOC reduces ocean carbon uptake and increases the social cost of carbon

**Felix Schaumann and Eduardo Alastrué de Asenjo**

**Felix Schaumann.**
**E-mail: felix.schaumann@uni-hamburg.de**

### This PDF file includes:

Figs. S1 to S5
Tables S1 to S4
SI References

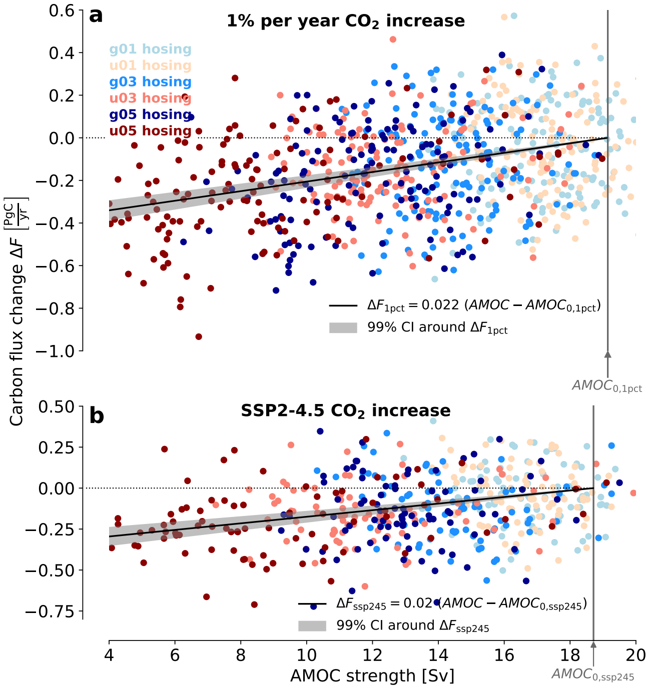


**(a)** **no** running mean

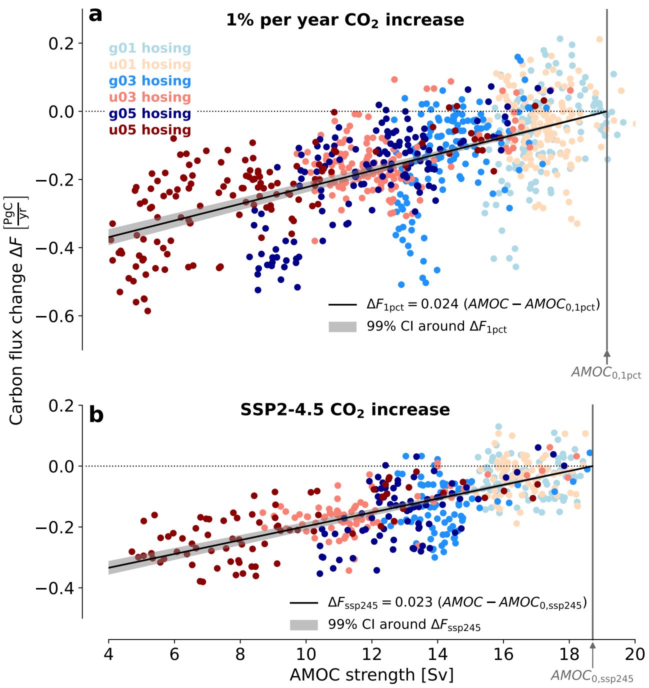


**(b)** running mean window: **5 years**

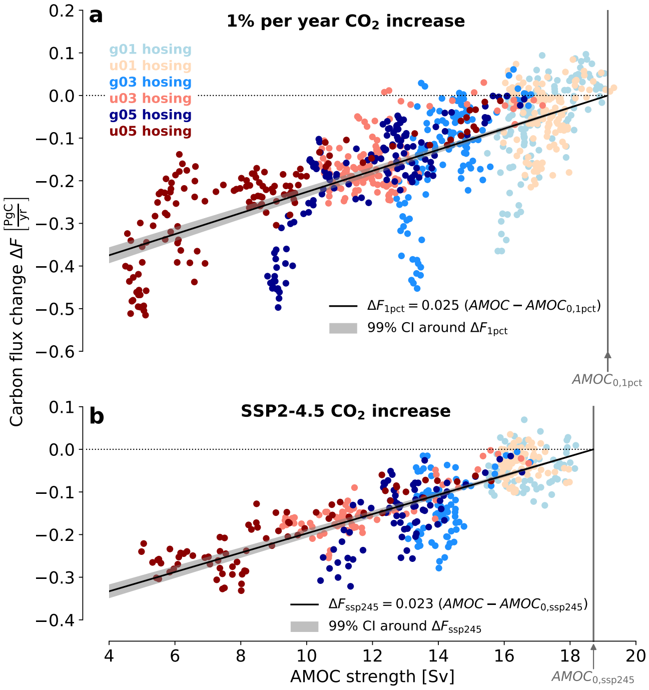


**(c)** running mean window: **10 years**

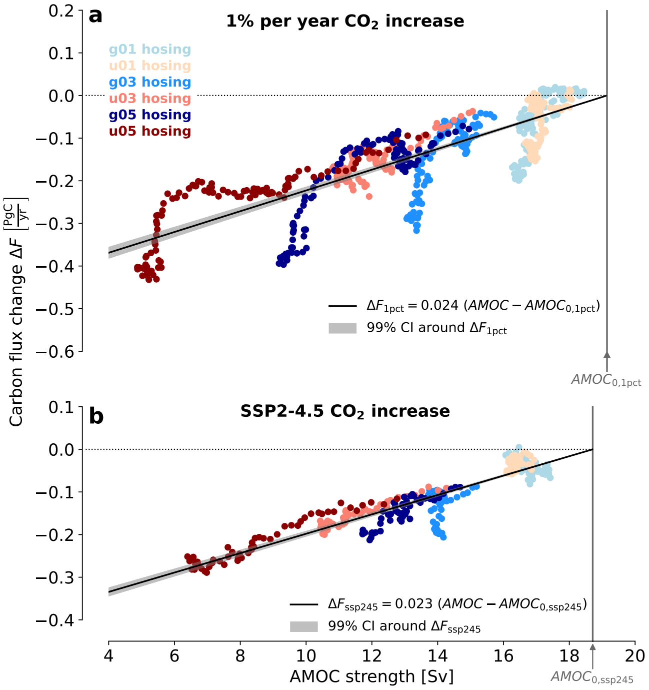


**(d)** running mean window: **30 years**

**Fig. S1.** Each panel shows a version of Fig. 3 in the paper, but for different running mean windows. The main specification is a running mean window of 10 years, such that panel **(c)** corresponds exactly to Fig. 4. The same running mean is applied to both the AMOC strength and the carbon storage time series. Note that panels **(a)** and **(b)** have wider y-axes due to higher fluctuations in the unsmoothed data. The regression coefficients, as plotted in each panel for both 1pct experiment and SSP2-4.5 scenario, are recalculated in for each running mean window size and are approximately constant across the different running mean configurations.

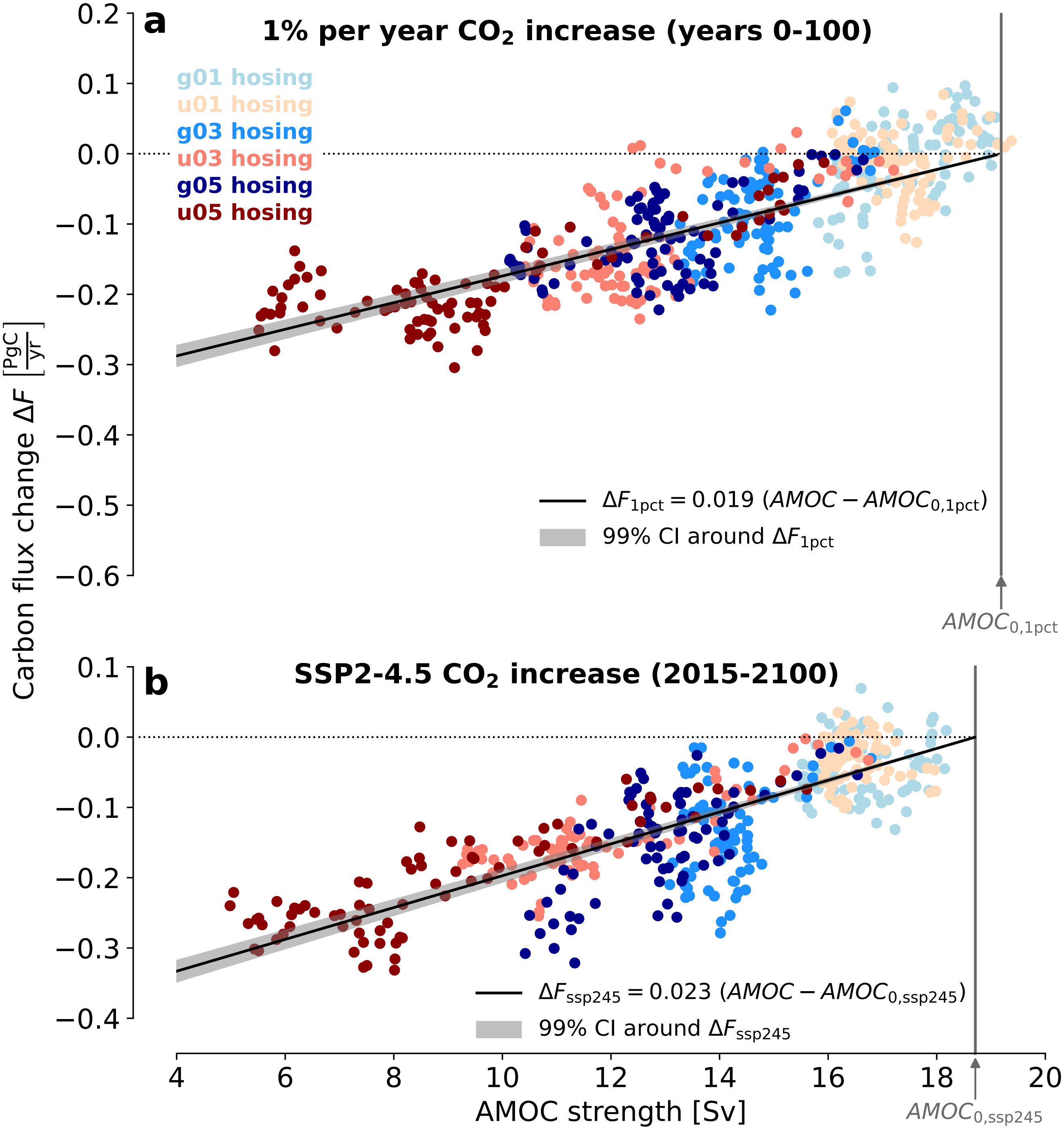


**Fig. S2.** A version of Fig. 3 in the paper, but omitting all data points of the 1pct experiment between year 100 and year 140. The reason for this robustness check is that between years 100 and 140, most 1pct hosing simulations show a substantial increase in carbon flux reductions (see also Fig. 2 in the paper). When omitting these 40 years of simulations, the linear regression coefficient weakens by around one quarter (0.019 rather than 0.025).

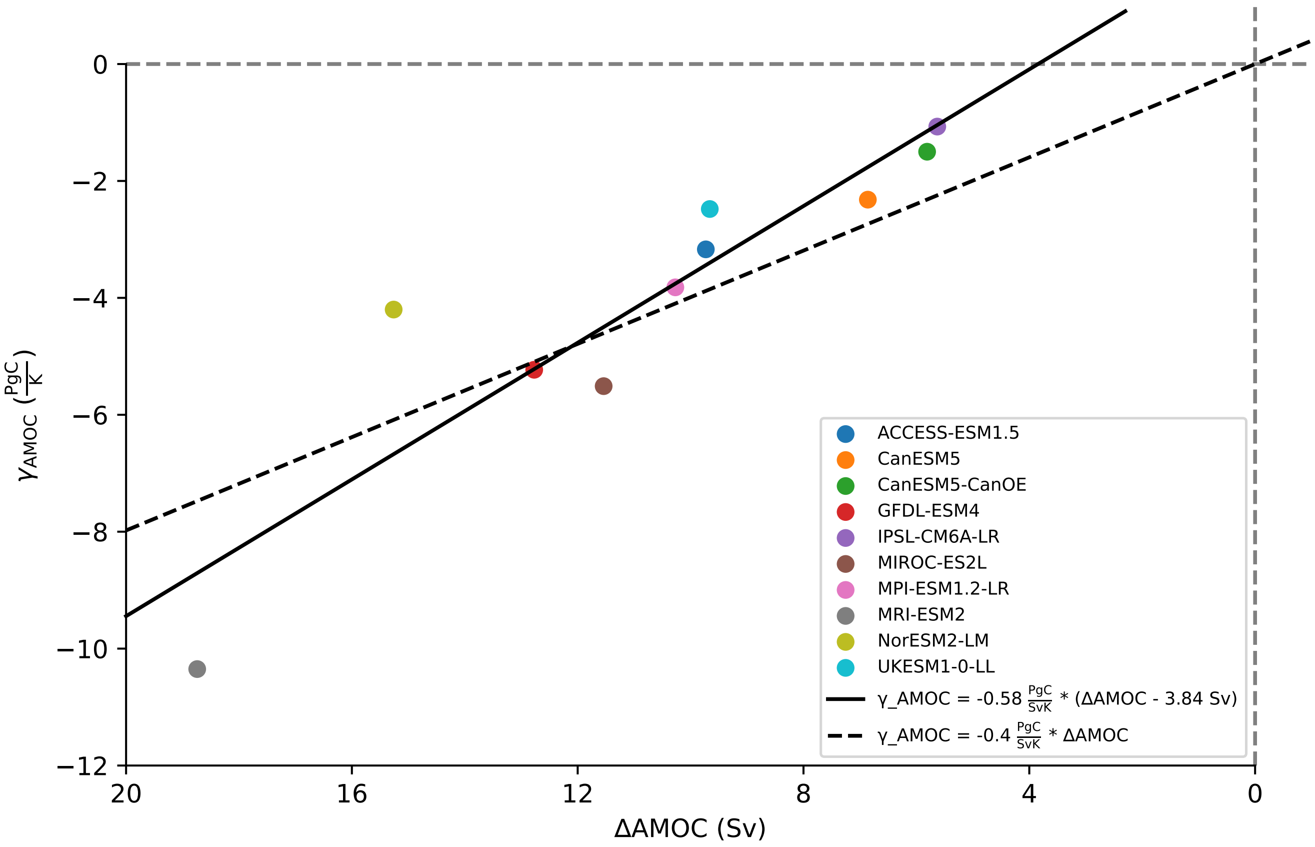


**Fig. S3.** AMOC-related carbon-climate feedback $\gamma_{\text{AMOC}}$ as a function of AMOC weakening $\Delta AMOC$. All values are taken from (1), and as in (1), the CNRM model is omitted due to unrealistic values for regenerated carbon. Two regression lines are estimated, the thick line as a linear linear regression with a constant order term, and the dashed line while forcing the intercept to be zero. For further calculations, we use the thick line which provides a better fit of the model data.

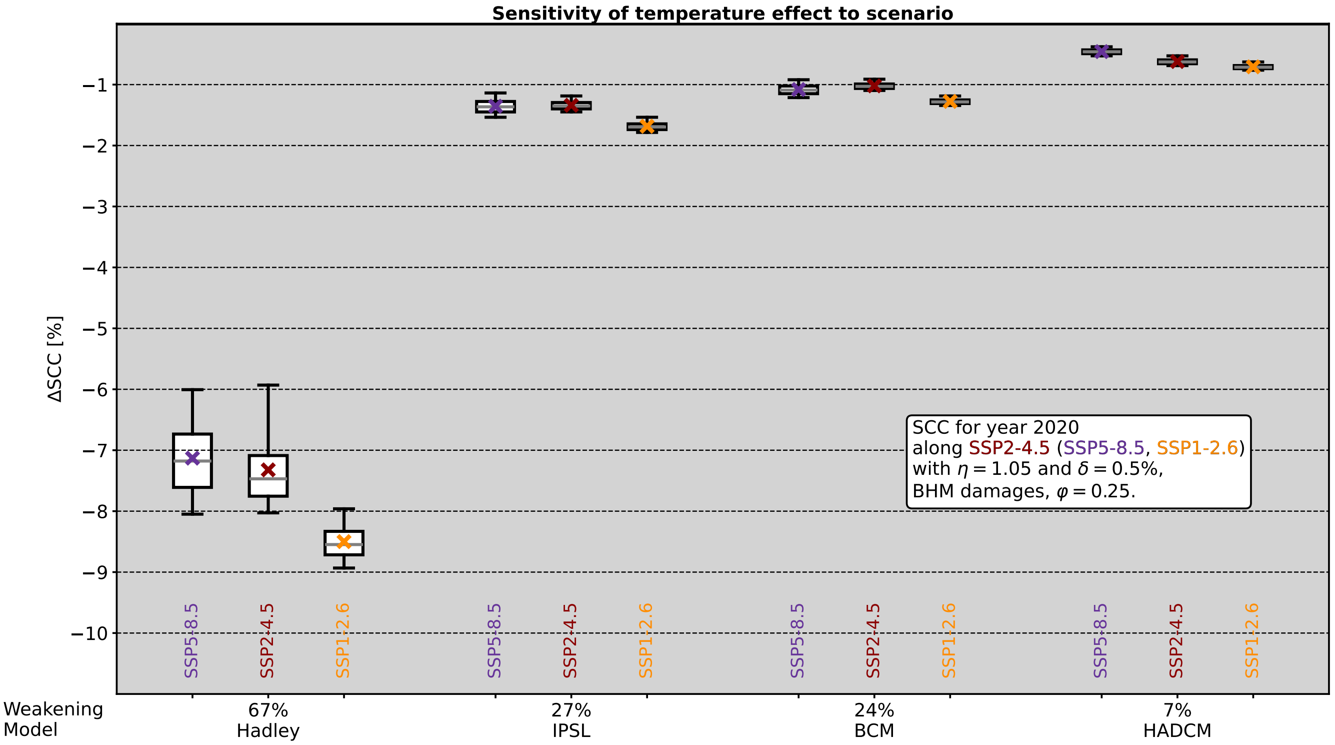


**Fig. S4.** A version of Fig. 5 in the paper, but focusing only on the SCC effects of AMOC-induced temperature pattern changes in META (2). For each climate model calibration, three different SSP scenarios are run for 1,000 Monte Carlo samples. The qualitative dependence of SCC changes on scenarios is the same as for the AMOC carbon feedbacks (effects are less/more pronounced in the SSP5-8.5/SSP1-2.5 scenario), but the dependence is weaker in the case of the temperature effect.

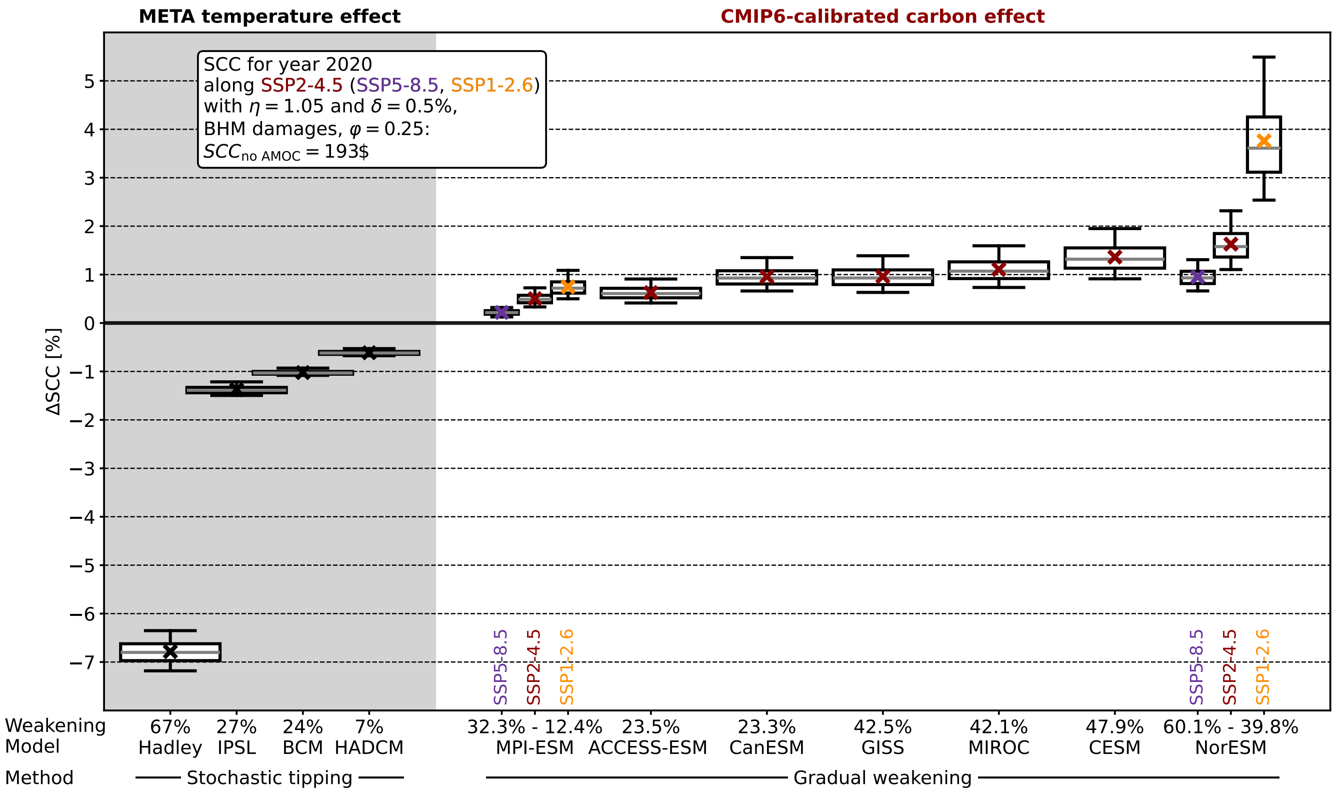


**(a)** A version of Fig. 5 in the paper with 0.1% of the most extreme Monte Carlo values trimmed on each tail of the distribution.

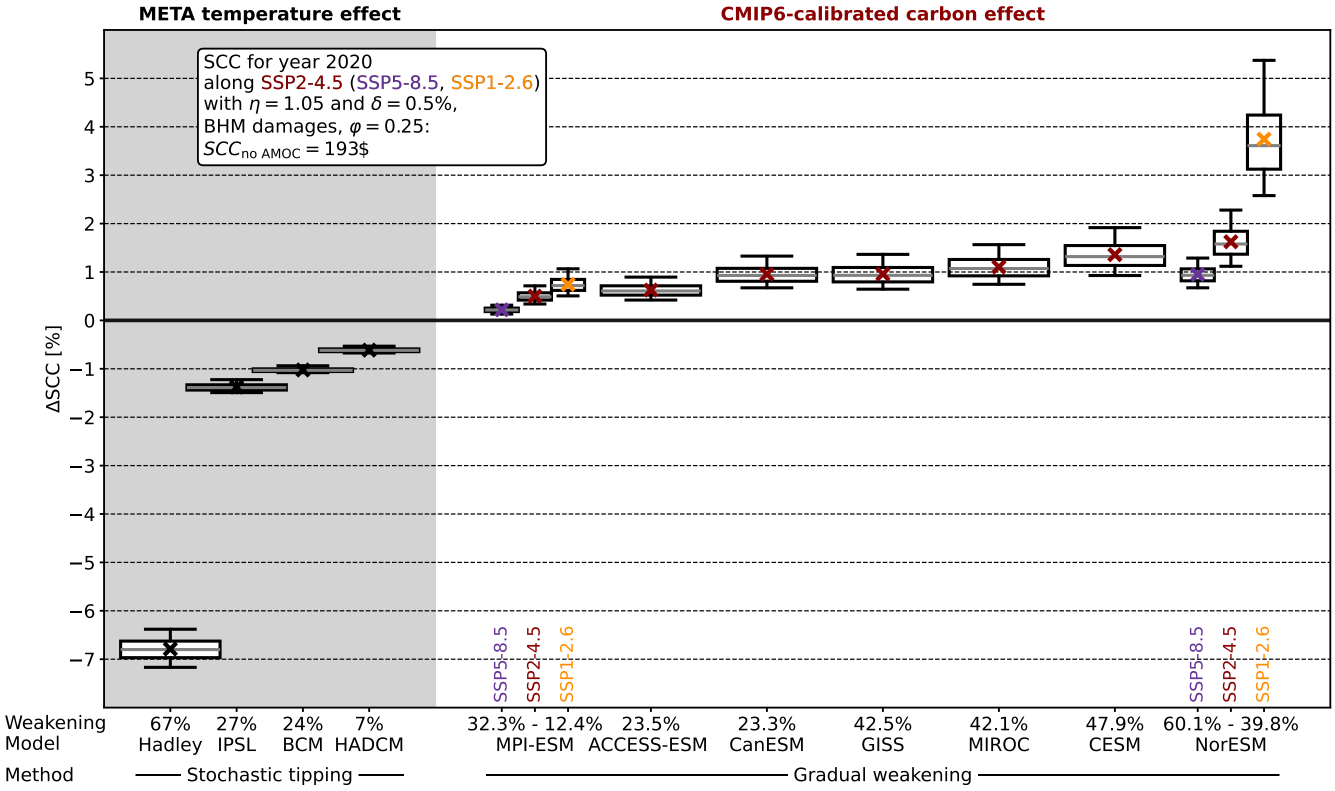


**(b)** A version of Fig. 5 in the paper with 1% of the most extreme Monte Carlo values trimmed on each tail of the distribution.

**Fig. S5.** The effect of trimming the tails of Monte-Carlo-generated SCC distributions. Fig. 5 in the paper shows the distribution of all 10,000 Monte Carlo samples. In order to test the robustness of these results to trimming extreme values at the tails of the distribution, we here show two additional versions of Fig. 5 with trimmed distributions. Panel **(a)** shows trimming of the 0.1% most extreme values at each tail; panel **(b)** shows trimming of the 1% most extreme values at each tail.

**Table S1. BGC-only simulations conducted in MPI-ESM1.2-LR.**

| Atm. $CO_2$ concentration | Hosing period | Hosing pattern | Hosing strength | Label |
|---|---|---|---|---|
| 1% per year $CO_2$ increase | — | — | — | no hosing |
| 1% per year $CO_2$ increase | year 0 - 140 | uniform in North Atlantic | 0.1 Sv | u01 hosing |
| 1% per year $CO_2$ increase | year 0 - 140 | uniform in North Atlantic | 0.3 Sv | u03 hosing |
| 1% per year $CO_2$ increase | year 0 - 140 | uniform in North Atlantic | 0.5 Sv | u05 hosing |
| 1% per year $CO_2$ increase | year 0 - 140 | decaying around Greenland | 0.1 Sv | g01 hosing |
| 1% per year $CO_2$ increase | year 0 - 140 | decaying around Greenland | 0.3 Sv | g03 hosing |
| 1% per year $CO_2$ increase | year 0 - 140 | decaying around Greenland | 0.5 Sv | g05 hosing |
| SSP2-4.5 scenario | — | — | — | no hosing |
| SSP2-4.5 scenario | 2015 - 2100 | uniform in North Atlantic | 0.1 Sv | u01 hosing |
| SSP2-4.5 scenario | 2015 - 2100 | uniform in North Atlantic | 0.3 Sv | u03 hosing |
| SSP2-4.5 scenario | 2015 - 2100 | uniform in North Atlantic | 0.5 Sv | u05 hosing |
| SSP2-4.5 scenario | 2015 - 2100 | decaying around Greenland | 0.1 Sv | g01 hosing |
| SSP2-4.5 scenario | 2015 - 2100 | decaying around Greenland | 0.3 Sv | g03 hosing |
| SSP2-4.5 scenario | 2015 - 2100 | decaying around Greenland | 0.5 Sv | g05 hosing |

**Table S2. Regressions of carbon flux changes on AMOC strength.**

| Name | Intercept $\left[\frac{\text{Pg}}{\text{yr}}\right]$ | Slope $c_0/c_2$ $\left[\frac{\text{Pg}}{\text{yr Sv}}\right]$ | Std. error of slope $\left[\frac{\text{Pg}}{\text{yr Sv}}\right]$ |
|---|---|---|---|
| $\Delta F_{0,\ \text{ssp245}}$ | 0 by definition | 0.023 | 0.0004 |
| $\Delta F_{1,\ \text{ssp245}}$ | -0.0013 | 0.022 | 0.0008 |
| $\Delta F_{0,\ \text{1pct}}$ | 0 by definition | 0.025 | 0.0004 |
| $\Delta F_{1,\ \text{1pct}}$ | 0.010 | 0.026 | 0.0008 |

See Methods for underlying regression equations.

**Table S3. Sensitivity analysis with respect to modified surface temperature patterns.**

| AMOC projection | ΔSCC pattern scaling | ΔSCC Hadley pattern | ΔSCC IPSL pattern | ΔSCC BCM pattern | ΔSCC HADCM pattern |
|---|---|---|---|---|---|
| MPI-ESM1.2-LR | 0.50 % | 0.51 % | 0.50 % | 0.50 % | 0.51 % |
| ACCESS-ESM1-5 | 0.63 % | 0.63 % | 0.63 % | 0.63 % | 0.64 % |
| CanESM5 | 0.95 % | 0.95 % | 0.95 % | 0.95 % | 0.95 % |
| GISS-E2-1-G | 0.96 % | 0.96 % | 0.96 % | 0.96 % | 0.97 % |
| MIROC-ES2L | 1.11 % | 1.11 % | 1.11 % | 1.11 % | 1.12 % |
| CESM2 | 1.36 % | 1.36 % | 1.36 % | 1.36 % | 1.37 % |
| NorESM2-LM | 1.63 % | 1.63 % | 1.63 % | 1.63 % | 1.64 % |

This table reports SCC results with the deterministic META model for all seven CMIP6-calibrated AMOC projections. The second columns report the SCC values as they are in the last column of Table 1, with the only difference being that they use the deterministic META model and not the mean of 10,000 Monte Carlo runs. The remaining four columns show the same quantity, but with modified pattern scaling. Given that AMOC weakening is expected to alter surface temperature patterns, we test the robustness of the carbon-caused SCC effect on the assumption of AMOC-induced temperature patterns. The second columns assumes that AMOC weakening doesn't influence the pattern scaling, whereas the other four columns overlay the AMOC-induced changes in surface temperature patterns used in (3) and (2). Every prescribed temperature pattern change starts in 2015, is linearly phased in until the year 2050, and stays constant after that. As the results show, the SCC effect of the AMOC carbon feedback does not depend on the specific assumption on how pattern scaling develops in this century.

**Table S4. Sensitivity analysis with respect to discounting and damage function parameters.**

| $\eta$ | $\delta$ | $\varphi$ | Damage function calibration | Additional damages (MPI-ESM \| NorESM) | Absolute SCC (MPI-ESM \| NorESM) | SCC change (MPI-ESM \| NorESM) |
|---|---|---|---|---|---|---|
| 0.8 | 0.1% | 0.25 | central | 12.3 trillion $ \| 27.8 trillion $ | 384.7 $ \| 390.4 $ | 0.67 % \| 2.19 % |
| 0.8 | 0.5% | 0.25 | central | 7.3 trillion $ \| 16.0 trillion $ | 261.8 $ \| 265.3 $ | 0.61 % \| 1.97 % |
| 0.8 | 1.0% | 0.25 | central | 3.8 trillion $ \| 8.2 trillion $ | 171.1 $ \| 173.1 $ | 0.52 % \| 1.70 % |
| 1.05 | 0.1% | 0.25 | central | 6.6 trillion $ \| 14.6 trillion $ | 277.9 $ \| 281.7 $ | 0.61 % \| 1.98 % |
| 1.05 | 0.5% | 0.25 | central | 3.9 trillion $ \| 8.5 trillion $ | 197.3 $ \| 199.7 $ | 0.54 % \| 1.75 % |
| 1.05 | 1.0% | 0.25 | central | 2.2 trillion $ \| 4.5 trillion $ | 135.8 $ \| 137.2 $ | 0.46 % \| 1.48 % |
| 1.5 | 0.1% | 0.25 | central | 2.2 trillion $ \| 4.8 trillion $ | 194.5 $ \| 196.7 $ | 0.44 % \| 1.58 % |
| 1.5 | 0.5% | 0.25 | central | 1.4 trillion $ \| 2.9 trillion $ | 148.8 $ \| 150.2 $ | 0.38 % \| 1.34 % |
| 1.5 | 1.0% | 0.25 | central | 0.8 trillion $ \| 1.6 trillion $ | 111.6 $ \| 112.5 $ | 0.33 % \| 1.13 % |
| 1.05 | 0.5% | 1.0 | low | 0.7 trillion $ \| 1.5 trillion $ | 30.0 $ \| 30.4 $ | 0.57 % \| 1.83 % |
| 1.05 | 0.5% | 1.0 | central | 1.2 trillion $ \| 2.7 trillion $ | 54.0 $ \| 54.7 $ | 0.60 % \| 1.91 % |
| 1.05 | 0.5% | 1.0 | high | 1.8 trillion $ \| 3.9 trillion $ | 78.4 $ \| 79.5 $ | 0.62 % \| 1.95 % |
| 1.05 | 0.5% | 0.25 | low | 2.3 trillion $ \| 5.0 trillion $ | 112.1 $ \| 113.5 $ | 0.53 % \| 1.72 % |
| 1.05 | 0.5% | 0.25 | central | 3.9 trillion $ \| 8.5 trillion $ | 197.3 $ \| 199.7 $ | 0.54 % \| 1.75 % |
| 1.05 | 0.5% | 0.25 | high | 5.2 trillion $ \| 11.5 trillion $ | 280.2 $ \| 283.5 $ | 0.54 % \| 1.75 % |
| 1.05 | 0.5% | 0.0 | low | 7.9 trillion $ \| 14.9 trillion $ | 2142.2 $ \| 2162.7 $ | 0.45 % \| 1.41 % |
| 1.05 | 0.5% | 0.0 | central | -1.0 trillion $ \| -6.5 trillion $ | 4078.2 $ \| 4119.2 $ | 0.49 % \| 1.50 % |
| 1.05 | 0.5% | 0.0 | high | -307.2 trillion $ \| -772.0 trillion $ | 6136.8 $ \| 6196.3 $ | 0.47 % \| 1.45 % |

$\eta$ is the marginal elasticity of intertemporal substitution; $\delta$ is the rate of pure time preference; $\varphi$ is the persistence parameter (0 corresponds to full persistence, 1 corresponds to no persistence); the damage function calibration is taken from (2) and represents the Burke-Hsiang-Miguel damage function (4) for "central" values, while "low" and "high" values refer to the boundaries of the 95% confidence interval of the coefficients from (4). Additional damages and SCC change are obtained like the values in Table 1 of the paper. SCC values are calculated for the year 2020 along the SSP2-4.5 scenario and given in 2010 USD. The AMOC is calibrated to MPI-ESM and NorESM projections, accordingly, because these two calibrations bound our impact estimates and serve to show the most extreme possible effects of a sensitivity analysis.

## References


1. A Katavouta, RG Williams, Ocean carbon cycle feedbacks in CMIP6 models: contributions from different basins. *Biogeosciences* **18**, 3189–3218 (2021).
2. S Dietz, J Rising, T Stoerk, G Wagner, Economic impacts of tipping points in the climate system. *Proc. Natl. Acad. Sci.* **118** (2021).
3. D Anthoff, F Estrada, RSJ Tol, Shutting Down the Thermohaline Circulation. *Am. Econ. Rev.* **106**, 602–606 (2016).
4. M Burke, SM Hsiang, E Miguel, Global non-linear effect of temperature on economic production. *Nature* **527**, 235–239 (2015).
